# How Does Problematic Internet Use Influence Chinese Rural Adolescent Externalizing Problem Behaviors? The Mediating Role of Mental Health and the Moderating Role of Parental Knowledge

**DOI:** 10.3390/ijerph20032162

**Published:** 2023-01-25

**Authors:** Shuping Yang, Xingchen Zhu

**Affiliations:** School of Education, Liaoning Normal University, Dalian 116029, China

**Keywords:** problematic Internet use, externalizing problem behaviors, mental health, parental knowledge, rural areas

## Abstract

This study aims to provide a new perspective on the relationship between problematic Internet use and externalizing problem behavior among adolescents. Many studies have focused on the relationship between problematic Internet use and adolescent externalizing problem behavior; however, research on the underlying mechanism remain understudied. Altogether, 1161 Chinese rural adolescents aged 13–17 years completed a self-report questionnaire. The results indicate that: (1) Problematic Internet use has a negative effect on rural adolescent externalizing problem behaviors; (2) mental health has an effect on the relationship between problematic Internet use and externalizing problem behaviors; and (3) parental knowledge may moderate the indirect effect by attenuating the relationship between problematic Internet use and externalizing problem behaviors. Regarding these findings, this study has discussed the potential reasons and provided some practical suggestions to improve problematic Internet use among rural adolescents.

## 1. Introduction

By the end of 2021, global Internet users have reached about 4.66 billion, an increase of 7.3% over the previous year [1]. The China Internet Network Information Center indicated that, as of December 2021, the number of Internet users in China had reached 1.032 billion, of which young Internet users under the age of 19 years accounted for 17.6%, reaching 182 million [2]. However, the prevalence of Internet addiction among Chinese adolescents is about 10.4%, which is much higher than the global overall rate of 6% [3]. Internet-related problems (e.g., problematic Internet use) have become a public health issue in China [4], and the Internet addiction rate is still on the rise [4].

Problematic Internet use is a type of online behavior that negatively affects psychological, social, school, and/or work situations [5]. For adolescents, Internet space is rich and colourful and allows for access to all kinds of information and fascinating games, which meet adolescents’ strong curiosity and thirst for knowledge [6,7]. Additionally, adolescents are a significant part of the online environment, and they are willingly involved in creating web content [8]. The Internet is an integral part of adolescents’ daily lives, which makes them the most significant group at risk for problematic Internet use [9,10]. Problematic Internet use has become a serious problem for adolescents [3,11,12], along with a range of problem behaviors [13,14].

Problem behavior refers to abnormal behavior exhibited by individuals that hinders their social adjustment [15]. Problem behaviors include internalizing problem behaviors such as anxiety and depression [16], and externalizing problem behaviors such as truancy, fighting, alcohol abuse, and aggression [17,18]. Self-determination theory states that the individual is affected by the social environment [19] and the Internet is an important social factor influencing adolescents’ development [20]. In line with this, social cognitive theory highlights that personal behavior is a product of the dynamic interaction between the external environment, internal subject factors, and past and present behavior [21]. Thus, Internet use is likely to lead to externalizing problem behaviors in adolescents. The current study provides some evidence that problematic Internet use as an external environmental factor can have a negative impact on adolescents’ daily work, academic performance, family relationships and mood (e.g., [22,23,24,25,26]). Several studies have also shown a clear association between problematic Internet use and behavioral problems (e.g., [13,27,28]).

The direct impact of problematic Internet use on adolescent problem behaviors has been studied. However, relevant research on the mechanism of the impact of problematic Internet use on Chinese rural adolescence externalizing problem behaviors is still insufficient. The detection rate of problem behaviors was much higher among Chinese rural adolescents than urban adolescents, and rural adolescents were more likely to exhibit problem behaviors compared to urban adolescents [29]. Additionally, as the 2020 national research report on minors’ Internet use of China shows, although the gap between urban and rural minors in terms of Internet access has basically disappeared, the two groups still significantly differ in Internet applications. Urban minor netizens (Internet users) more commonly use the Internet for information acquisition and interpersonal communication, whereas rural minor netizens more often limit their Internet use to entertainment purposes [30]. Several studies also found that rural adolescents exhibit more problematic Internet use than urban adolescents [7,31,32]. As such, it is critical to explore protective factors at the environmental and individual levels to promote Chinese rural adolescent behaviors. Moreover, there is also a lack of empirical research focused on rural adolescents from China. Therefore, during adolescence, the association between problematic Internet use and Chinese rural adolescent externalizing problem behaviors remains to be investigated and unveiled.

### 1.1. Mental Health as a Mediator

Mental health is considered a protective factor against problem behaviors in adolescents [33]. Studies have shown that poor mental health is a major cause of problem behaviors [34]. For example, Wu [35] found that mental health could influence adolescent problem behaviors, and poor mental health were an important trigger for these behaviors. Chen and Zhong [36] also found that poor mental health, such as stress, depression, or feeling worthless, had a significant negative effect on adolescent problem behaviors, such as smoking, drinking, and fighting.

In addition, mental health problems may be related to conditions or behaviors that involve problematic Internet use [37], as problematic Internet use develops at the expense of an individual’s physical, emotional, and (importantly) mental health [38]. Previous studies have also shown a negative correlation between problematic Internet use and some indicators of mental health, such as subjective well-being and vitality [39,40].

Regarding the mechanism among problematic Internet use, mental health and adolescent externalizing problem behaviors, mental health may possibly produce the intermediary effect between problematic Internet use and externalizing problem behaviors.

### 1.2. Parental Knowledge as a Moderator

Even though problematic Internet use serves as a general risk factor for externalizing behavior problems, many adolescents tend to have more resilient outcomes than others. According to the risk-buffering model [41,42], protective factors are particularly beneficial in neutralizing the negative effects of risk. When protective factors are present, the negative impact of adversity on adolescent development can be effectively reduced. However, if such protective factors are not present or are no longer present, the predictive effect of risk factors on negative development is greatly enhanced [41,42]. Consequently, when examining the relation between problematic Internet use and externalizing problem behaviors, protective factors should be taken into consideration. Parental knowledge, also referred to as parental behavioral control or parental monitoring. It generally refers to a set of interrelated parental behaviors related to caring for and keeping track of children’s activities, associates, friends, and whereabouts [43]. In fact, the influence of parental knowledge on adolescent behavioral development has attracted increasing attention [44,45,46].

The individual is not isolated but is embodied within a social environment. Educational ecosystem theory highlights the role of the family in promoting the development of adolescents [47]. Indeed, parental knowledge is a powerful protective factor that helps buffer the impact of risky environments and reduces the emergence of problem behaviors in adolescents [45,48,49]. Furthermore, some studies have proven that parental knowledge can decrease adolescent problematic Internet use [50,51], and externalizing problem behaviors [44,52,53]. Parental knowledge is developed in close and trusting parent-child relationships, which may be the best process for reducing adolescent problem behaviors [54,55,56]. Adolescents with higher levels of parental knowledge may experience greater resilience in the face of adversity [45,57].

Regarding the mechanism among problematic Internet use, parental knowledge and adolescent externalizing problem behaviors, parental knowledge may possibly produce the moderating effect between problematic Internet use and adolescent externalizing problem behaviors.

### 1.3. The Present Study

As the occurrence of adolescent externalizing problem behaviors might be influenced by factors across domains (including Internet, family and individuals), it is difficult to address and prevent adolescent externalizing problem behaviors from a single domain. The findings mentioned above suggest that problematic Internet use may be a salient predictor of adolescent externalizing problem behaviors, and mental health may possibly produce the intermediary effect between problematic Internet use and externalizing problem behaviors and parental knowledge may play an important role in buffering the association between problematic Internet use and adolescent externalizing problem behaviors. The present exploration of these factors contributes to the construction of a meaningful intervention plan for adolescent externalizing problem behaviors, which is a matter that has seldom been given attention in the existing literature—to the best of our knowledge, no previous research has explored the combined influences of problematic Internet use, mental health and parental knowledge on externalizing problem behaviors among adolescents. Therefore, based on the self-determination theory, social cognitive theory, educational ecosystem theory and risk-buffering model, this study expands the existing literature on problematic Internet use and externalizing problem behaviors.

**Figure 1:**
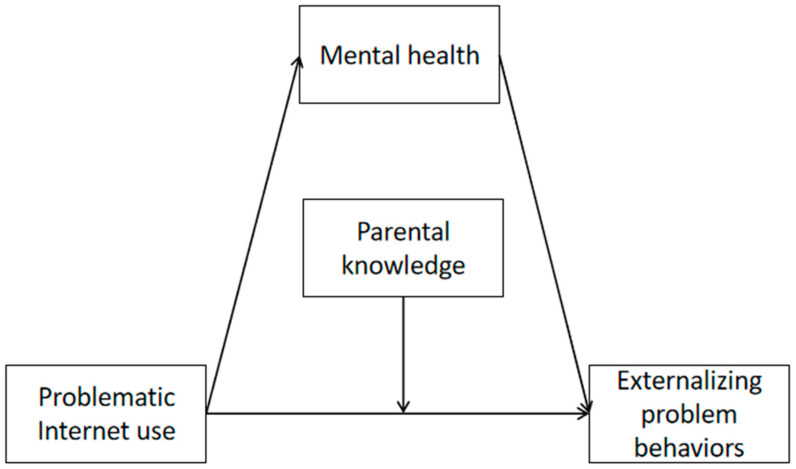
A hypothesized moderated mediation model.

To this end, we propose the hypotheses from H1 to H3 (See Figure 1):

**Hypothesis 1 (H1).**  Problematic Internet use has a negative effect on rural adolescent externalizing problem behaviors.

**Hypothesis 2 (H2).**  Mental health mediates the relationship between problematic Internet use and externalizing problem behaviors among rural adolescents.

**Hypothesis 3 (H3).**  Parental Knowledge modifies the effect of problematic Internet use on externalizing problem behaviors among rural adolescents.

## 2. Materials and Methods

### 2.1. Participants

A total of 1196 participants were recruited from four junior high schools in five counties of Liaoning Province, China by stratified and random cluster sampling. Students completed the pencil and paper-based survey in the classroom during school hours under the supervision of their teachers and research assistants. After removing 35 invalid questionnaires which were found to have many missing contents through screening, a total of 1161 questionnaires were obtained, with a response rate of 97%. Participants ranged from 13 to 17 years of age (M = 14.45, SD = 0.630), of which 50.6% (N = 587) were boys and 49.4% (N = 574) were girls. Data collection occurred at the end of 2021. Permission was obtained from the schools. Written informed consent was obtained from all participants and their parents or guardians. The informed consent form did not require a signature or the students’ name and we guaranteed anonymity and confidentiality. Students were told that the participation was completely voluntary and they could withdraw from the study at any time. With the guidance of trained research assistants, students who agreed to participate in the questionnaire survey completed the questionnaire independently in the classroom during school hours. Students completed the questionnaire, put it in an envelope and sealed it. A small gift (i.e., a notebook; a chocolate) was offered for their participation. All research materials in this study were reviewed and approved by the authors’ university research ethics committee.

### 2.2. Measures

Externalizing problem behaviors. Externalizing problem behaviors was measured by the Youth Self-Report (YSR, [58,59]), which have been proved to be reliable and valid in previous studies [60,61]. The YSR Externalizing Problem Behavior Scale consists of two subscales (aggressive behavior and rule-breaking behavior) with a total of 30 items. An example item is “I destroy my own things”. Items were rated on a 3-point Likert scale ranging from 1 (not true) to 3 (very true). Through calculating the average of thirty items, a higher score indicated a higher level of adolescent externalizing problem behaviors. Additionally, this measure showed good internal reliability, with a Cronbach’s alpha of 0.926.

Problematic Internet use. Problematic Internet use was measured using a ten-item scale based on the Chinese version of the PIU Questionnaire [62], the items of which were selected from Internet Dependency Questionnaire [63]. This measure has demonstrated good reliability and validity in Chinese adolescents [11,64]. An example item is “Have you made unsuccessful efforts to control, cut back, or stop Internet use”. Items were rated on a 6-point Likert scale ranging from 1 (not at all true) to 6 (always true). Through calculating the average of all items, a higher score indicated a higher level of problematic Internet use. Additionally, this measure showed good internal reliability, with a Cronbach’s alpha of 0.961.

Mental health. Mental health was measured using the 12-item General Health Questionnaire (GHQ-12) [65], which has been widely used among Chinese adolescents (e.g., [66]). It consists of 12 items, half positive (e.g., “able to concentrate on whatever I do”) and half negative (e.g., “insomnia due to anxiety”), with a 4-point Likert scale ranging from 1 (never) to 4 (often). The scores of items on negative mental health were calculated in a reverse order. Through calculating the average of all items, a higher score indicated better mental health. Additionally, this measure showed good internal reliability, with a Cronbach’s alpha of 0.917.

Parental Knowledge. Parental Knowledge was measured using the 5-item scale based on the parental monitoring questionnaire [54], which demonstrated good reliability and validity for the Chinese adolescents [44,45]. An example item is, “Do your parents know what activities you do in your free time”. Items were rated on a 3-point Likert scale ranging from 1 (know little) to 3 (know much). Through calculating the average of all items, a higher score indicated a higher level of parental knowledge. Additionally, this measure showed acceptable internal reliability, with a Cronbach’s alpha of 0.777.

Control variables. Based on previous studies [44,62,67,68], two categories of control variables related to adolescent externalizing problem behaviors were chosen for this study. 

Family characteristics. Family characteristics include the family’s book collection, father’s education, mother’s education, parents’ educational expectations, and family’s economic conditions. Based on the question “Do you have many books in your family (excluding textbooks and magazines)?”, family’s book collection responses were divided into five categories. The response options for each topic were assigned the following scores: 1 = few, 2 = relatively few, 3 = average, 4 = relatively many, and 5 = many. According to the questions, “The educational level of your father is” and “The educational level of your mother is”, the response options for each topic were assigned the following scores: 1 = no education, 2 = primary school, 3 = junior high school, 4 = technical secondary school/technical school, 5 = vocational high school, 6 = ordinary high school, 7 = junior college, 8 = bachelor’s degree, and 9 = master’s degree and above. Based on the question “What education level would your parents like you to be equipped with?”, parental education expectation responses were divided into nine categories. The response options for each topic were assigned the following scores: 1 = drop out of school now, 2 = junior high school, 3 = technical secondary school/technical school, 4 = vocational high school, 5 = ordinary high school, 6 = junior college, 7 = bachelor’s degree, 8 = master’s degree, and 9 = PhD. Family economic conditions was measured based on the question, “What are the current economic conditions in your family?”. The response to each topic were assigned the following scores: 1 = very difficult, 2 = relatively difficult, 3 = medium, 4 = relatively rich, and 5 = best.

Individual characteristics. We further control the individual adolescent’s characteristics, including gender, nationality, age and physical health condition.

### 2.3. Statistical Analysis

SPSS Version 23.0 and STATA Version 16.0 were used for data analysis. First, descriptive statistics and bivariate correlations were used to assess the relationship among core variables. Second, when analyzing the impact of problematic Internet use on adolescent externalizing problem behaviors, the method of gradually increasing influencing factors was used for the OLS (Ordinary Least Squares) regression results. Next, the significance of the mediation effect was tested by bootstrapping the 95% confidence interval (95% CIs) of the indirect and direct effect with 5000 repetitions. Specifically, the 95% confidence interval that does not include zero provides evidence for a significant indirect and direct effect. Finally, moderated mediation analyses were performed using the PROCESS 4.0 macro for SPSS with covariates.

## 3. Results

### 3.1. Correlation Analysis of Variables

Bivariate correlations between the core variables were conducted by using Pearson correlation analysis. The results are presented in Table 1, including the mean values, SDs, and Pearson correlation values of the core variables. The results indicated that all core variables are significantly associated. Externalizing problem behaviors was positively associated with problematic Internet use (r = 0.230 *p* < 0.01), but negatively associated with Mental health (r = −0.371, *p* < 0.01) and parental knowledge (r = −0.253, *p* < 0.01). Moreover, problematic Internet use was negatively associated with Mental health (r = −0.167, *p* < 0.01) and parental knowledge (r = −0.095, *p* < 0.01). Finally, mental health was positively associated with parental knowledge (r = 0.188, *p* < 0.01).

### 3.2. OLS Regression Results

The results in Table 2 showed that problematic Internet use had a negative effect on adolescent externalizing problem behaviors. First, in model (1), only the independent variable problematic Internet use was introduced. Second, model (2) introduced family characteristics variables on the basis of model (1). Finally, model (3) introduced adolescent individual characteristic variables on the basis of model (2). In Table 2 models (1) to (3), the estimated coefficients of problematic Internet use was 0.062 (*p* < 0.01), 0.058 (*p* < 0.01) and 0.052 (*p* < 0.01), respectively, which passed the 1% significance test, indicating that problematic Internet use positively predicted adolescent externalizing problem behaviors.

### 3.3. Mediation Analyses

The results in Table 3 showed that mental health had an effect on the relationship between problematic Internet use and adolescent externalizing problem behaviors after controlling for control variables. To verify the significance of the mediating effect, this study used the non-parametric percentile bootstrap method. Mental health and peer relationship, regardless of direct or indirect effects, do not contain 0 in their 95% confidence intervals, which verified the significance of the mediation effect.

### 3.4. Moderated Analyses

The results in Table 4 showed that parental knowledge moderated the effect of problematic Internet use on externalizing problem behaviors. First, the estimated coefficients of problematic Internet use and was 0.044 (*p* < 0.01), which passed the 1% significance test. Second, the estimated coefficients of the interaction term of problematic Internet use and parental knowledge was −0.028 (*p* < 0.10), which also passed the 10% significance test.

**Figure 2:**
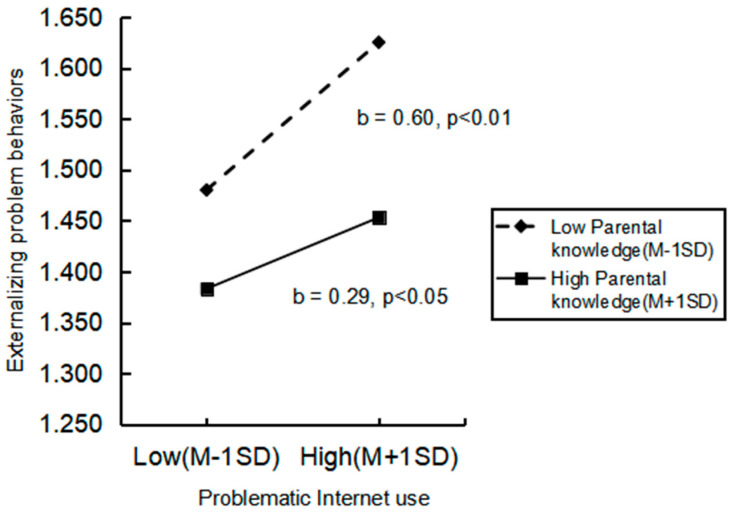
Simple slope analyses of the moderating effect of parental knowledge.

As shown in Figure 2, the nature of the moderation was further explored using a simple slope analysis and conditioned as low (−1 SD) and high (+1 SD). This analysis proved the effect to be significant for all groups. Specifically, compared with the individuals who had a higher level of parental knowledge, individuals with a lower level of parental knowledge tended to show a stronger negative relationship between problematic Internet use on externalizing problem behaviors.

## 4. Discussion

The findings from this study confirm the first hypothesis of the study (H1) by indicating that problematic Internet use played a negative role in promoting adolescent externalizing problem behaviors. This result is consistent with the research of Fineberg et al. [38] and Fontana et al. [13]. The likely reason is that problematic Internet use can be defined as a situation in which Internet users lose control over internet use despite recognizing the serious negative impact of such behavior on their daily lives [69,70]. Furthermore, the Internet offers objectionable content, including web pages containing age-inappropriate audiovisual material, violent video games, and unprotected chat rooms and discussion boards [71,72]. Thus, the longer adolescents use the Internet, the more likely they are to be exposed to undesirable information in the network and the more likely their behavior will be adversely influenced [73,74]. Another possible explanation is that as many rural parents migrate to cities for job opportunities, their rural children tend to receive less parental care and poorer attachment [75], which may also lead to excessive Internet use [76]. Additionally, Internet addiction increases the incidence of adolescent problem behaviors [77]. However, it is also important to note that adolescents with externalizing problem behaviors may have fewer friends in real life [78]. Therefore, when they are not accepted by their peers, they probably choose to escape and spend more time online with other players [79]. Thus, there may be a bidirectional relationship between problematic Internet use and adolescent externalizing problem behaviors.

Mental health is the intermediary mechanisms of problematic Internet use that affect the development of adolescent externalizing problem behaviors. This means that our second hypothesis (H2) has been confirmed by empirical evidence. Problematic Internet use can damage adolescents’ mental health, thereby increasing the frequency of their externalizing problem behaviors. One possible reason is that the negative nature of online activities can damage and distort the psychological development of adolescents [80,81,82]. Additionally, adolescents with poor mental health are more likely to develop externalizing problem behaviors [36]. These findings clarify the mechanism of the impact of problematic Internet use on adolescent externalizing problem behaviors, enabling better understanding of the laws affecting the development of adolescent externalizing problem behaviors, expanding the current relevant theories of adolescent behaviour development, and playing a practical theoretical guiding role in promoting the development of adolescents’ pro-social behaviour. It also supports the importance of mental health [34,83] among adolescents.

This study also confirmed the third hypothesis (H3), that is, parental knowledge plays a role by showing a moderating effect on the relationship between problematic Internet use and adolescent externalizing problem behaviors. According to educational ecosystem theory [47], adolescents with high levels of positive family characteristics, such as parental knowledge, are less likely to engage in problem behaviors in high-risk environments (e.g., problematic Internet use). The possible explanation is for families with high levels of parental knowledge, adolescents grow up in a more harmonious and warmer home where parents are likely to provide them with good guidance and therefore exhibit lower levels of problem behaviors [44,84]. This implies that when parents are less knowledgeable about their children’s activities and friendships, problematic Internet use can take a serious toll and eventually lead to higher levels of problem behaviors.

The results from this study have implications and contributions from both theoretical and practical perspectives. Regarding theoretical contributions, first, this study highlights the underlying mechanism of the impact of problematic Internet use on adolescent externalizing problem behaviors, which broadens and complements the existing empirical studies [13,38]. This study also extends our understanding of the relationship between mental health, parental knowledge and externalizing problem behaviors from societal perspectives.

Our findings also have practical implications. First, government should improve laws to strengthen the regulation of Internet use among adolescents. For example, the government should establish a corresponding quality certification mechanism to ensure that content producers provide high-quality products and provide appropriate entertainment games, search engines, and social tools for the characteristics and needs of adolescents. Additionally, government should also further promote the reform of the household registration system, weaken the restrictions on household registration, and allow parents who migrate to the cities to bring their children with them, so that rural adolescents can grow up with the company of their parents. Second, schools should use new technology to introduce interactive teaching and learning to guide rural adolescents to learn online instead of being addicted to online games. Third, parents should improve their children’s time management and self-management skills, supervise their children’s Internet use, and try to guide them to use the Internet wisely. Parents should also focus on cultivating children’ interests, increasing their time for outdoor activities after school, and reducing their dependence on the Internet.

## 5. Limitations

This study also has several limitations. First, the sample was only systematically collected in Liaoning Province, rather than a larger national sample. The generalizability of the findings should be further tested by obtaining a nationally representative sample. Second, the cross-sectional data only provide a snapshot between the factors of problematic Internet use and adolescent externalizing problem behaviors. Further research that takes into account longitudinal effects would be helpful in understanding the causal relationship between adolescent externalizing problem behaviors and correlates. Third, this study relied on a self-report survey that reflected only adolescents’ perspectives. To extend this study, future reports from multiple informants (e.g., parents, peers, and teachers) should be included.

## 6. Conclusions

This study examined rural adolescent externalizing problem behaviors and explored several antecedent factors that influence their externalizing problem behaviors. Specifically, this study found that problematic Internet use might have a negative impact on rural adolescent externalizing problem behaviors, and mental health could be a mediator within the relationship between problematic Internet use and rural adolescent externalizing problem behaviors. Additionally, this study found that parental knowledge could display a moderating effect between problematic Internet use and rural adolescent externalizing problem behaviors. This study has several theoretical and practical contributions. First, this study enriches the theoretical perspective between problematic Internet use and rural adolescent externalizing problem behaviors by highlighting the underlying mechanism of the impact of problematic Internet use on adolescent externalizing problem behaviors. Second, this study integrates social and individual perspectives to search for potential factors for improving rural adolescent externalizing problem behaviors, thus depicting a more comprehensive picture of rural adolescent development. Finally, the findings of this study provide some practical suggestions to government, schools and parents.

## Figures and Tables

**Table 1:** Correlation analysis between key variables.

	Mean	SD	Externalizing Problem Behaviors	Problematic Internet Use	Mental Health	Parental Knowledge
Externalizing problem behaviors	1.487	0.328	-			
Problematic Internet use	2.576	1.210	0.230 ***	-		
Mental health	2.841	0.718	−0.371 ***	−0.167 ***	-	
Parental knowledge	2.336	0.545	−0.253 ***	−0.095 ***	0.188 ***	-

Note. *** *p* < 0.01.

**Table 2:** OLS regression results for the effect of problematic Internet use on rural adolescent externalizing problem behaviors.

Variables	(1) Externalizing Problem Behaviors	(3) Externalizing Problem Behaviors	(4) Externalizing Problem Behaviors
Problematic Internet Use	0.062 *** (0.010)	0.058 *** (0.010)	0.052 *** (0.010)
Family’s book collection		−0.024 ** (0.010)	−0.019 * (0.010)
Father’s educational level		0.010 (0.007)	0.011 (0.007)
Mother’s educational level		−0.007 (0.007)	−0.007 (0.007)
Parental educational expectations		−0.024 *** (0.007)	−0.023 *** (0.007)
Family economic conditions		0.012 (0.019)	0.006 (0.019)
Age			−0.003 (0.015)
Gender			0.080 *** (0.018)
Nationality			0.038 *** (0.036)
Physical Health condition			−0.038 *** (0.010)
Sample size	1161	1161	1161
R2	0.053	0.078	0.102

Note. * p < 0.1, ** p < 0.05, *** p < 0.01. The robust standard errors are in parentheses.

**Table 3:** Mediation effect test results (bootstrap method).

Variables	Effect	Coefficient	Standard Error	95% Confidence Interval
Mental health	Indirect	0.014	0.003	[0.008–0.020]
	Direct	0.038	0.009	[0.020–0.056]

Note: Shown are 95% confidence intervals constructed using the bias-corrected and accelerated methods.

**Table 4:** Moderated regression analyses predicating rural adolescent externalizing problem behaviors.

Variables	Externalizing Problem Behaviors
Problematic Internet Use	0.044 *** (0.009)
Parental Knowledge	−0.124 *** (0.017)
Problematic Internet Use × Parental Knowledge	0.028 * (0.016)
Control variables	Yes
Sample size	1161
R2	0.147

Note. * p < 0.1, *** p < 0.01. The robust standard errors are in parentheses.

## Data Availability

The data used in this research are available on request from the corresponding author. The data are not publicly available due to restrictions.

## References

[1] Narmanov U. (2022). The role and importance of the digital economy in the development of innovative. Linguist. Cult. Rev..

[2] China Internet Network Information Center (2022). The 49th Statistical Report on the Development of China’s Internet. http://www.cnnic.cn/hlwfzyj/hlwxzbg/hlwtjbg/202202/P020220407403488048001.pdf.

[3] Wang H. (2022). The Effects of School Climate, Parent–Child Closeness, and Peer Relations on the Problematic Internet Use of Chinese Adolescents: Testing the Mediating Role of Self-Esteem and Depression. Int. J. Environ. Res. Public Health.

[4] Shao Y.J., Zheng T., Wang Y.Q., Liu L., Chen Y., Yao Y.S. (2018). Internet addiction detection rate among college students in the People’s Republic of China: A meta-analysis. Child Adolesc. Psychiatry Ment. Health.

[5] Beard K.W. (2005). Internet addiction: A review of current assessment techniques and potential assessment questions. CyberPsychology Behav..

[6] Colley A., Maltby J. (2008). Impact of the Internet on our lives: Male and female personal perspectives. Comput. Hum. Behav..

[7] Kormas G., Critselis E., Janikian M., Kafetzis D., Tsitsika A. (2011). Risk factors and psychosocial characteristics of potential problematic and problematic problematic Internet use among adolescents: A cross-sectional study. BMC Public Health.

[8] Tkáčová H., Pavlíková M., Jenisová Z., Maturkanič P., Králik R. (2021). Social media and students’ wellbeing: An empirical analysis during the COVID-19 pandemic. Sustainability.

[9] Faghani N., Akbari M., Hasani J., Marino C. (2020). An emotional and cognitive model of problematic Internet use among college students: The full mediating role of cognitive factors. Addict. Behav..

[10] Sela Y., Zach M., Amichay-Hamburger Y., Mishali M., Omer H. (2020). Family environment and problematic internet use among adolescents: The mediating roles of depression and fear of missing out. Comput. Hum. Behav..

[11] Zhang J., Jiao C., Yu C., Qiao T., Li Z. (2021). Heterogeneous Association of Chinese Adolescents’ Engaged Living with Problematic Internet Use: A Mixture Regression Analysis. Front. Psychol..

[12] Ni X., Qian Y., Wang Y. (2017). Factors affecting pathological Internet use among Chinese university students. Soc. Behav. Personal. Int. J..

[13] Fontana A., Benzi I.M.A., Cipresso P. (2022). Problematic internet use as a moderator between personality dimensions and internalizing and externalizing symptoms in adolescence. Curr. Psychol..

[14] Jaworska N., MacQueen G. (2015). Adolescence as a unique developmental period. J. Psychiatry Neurosci. JPN.

[15] Achenbach T.M., McConaughy S.H., Howell C.T. (1987). Child/adolescent behavioral and emotional problems: Implications of cross-informant correlations for situational specificity. Psychol. Bull..

[16] Lee A., Hankin B.L. (2009). Insecure attachment, dysfunctional attitudes, and low self-esteem predicting prospective symptoms of depression and anxiety during adolescence. J. Clin. Child Adolesc. Psychol..

[17] Petersen I.T., Bates J.E., Dodge K.A., Lansford J.E., Pettit G.S. (2015). Describing and predicting developmental profiles of externalizing problems from childhood to adulthood. Dev. Psychopathol..

[18] Brock R.L., Kochanska G. (2016). Interparental conflict, children’s security with parents, and long-term risk of internalizing problems: A longitudinal study from ages 2 to 10. Dev. Psychopathol..

[19] Reeve J., Ryan R.M., Deci E.L. (2018). Sociocultural influences on student motivation as viewed through the lens of self-determination theory. Big Theor. Revisit..

[20] Ahn J. (2011). The effect of social network sites on adolescents’ social and academic development: Current theories and controversies. J. Am. Soc. Inf. Sci. Technol..

[21] Bandura A. (1999). Social cognitive theory: An agentic perspective. Asian J. Soc. Psychol..

[22] Sha P., Sariyska R., Riedl R., Lachmann B., Montag C. (2019). Linking internet communication and smartphone use disorder by taking a closer look at the Facebook and WhatsApp applications. Addict. Behav. Rep..

[23] Leménager T., Hoffmann S., Dieter J., Reinhard I., Mann K., Kiefer F. (2018). The links between healthy, problematic, and addicted Internet use regarding comorbidities and self-concept-related characteristics. J. Behav. Addict..

[24] Ko C.H., Yen J.Y., Chen C.C., Chen S.H., Wu K., Yen C.F. (2006). Tridimensional personality of adolescents with internet addiction and substance use experience. Can. J. Psychiatry.

[25] Lin S.S., Tsai C.C. (2002). Sensation seeking and internet dependence of Taiwanese high school adolescents. Comput. Hum. Behav..

[26] Ryu E.J., Choi K.S., Seo J.S., Nam B.W. (2004). The relationships of Internet addiction, depression, and suicidal ideation in adolescents. J. Korean Acad. Nurs..

[27] Griffiths M.D., Parke J. (2002). The social impact of internet gambling. Soc. Sci. Comput. Rev..

[28] Beard K.W., Wolf E.M. (2001). Modification in the proposed diagnostic criteria for Internet addiction. Cyberpsychology Behav..

[29] Chen B.B., Qu Y., Yang B., Chen X. (2022). Chinese mothers’ parental burnout and adolescents’ internalizing and externalizing problems: The mediating role of maternal hostility. Dev. Psychol..

[30] The Youth Rights, Interests Protection Department of the Communist Youth League Central Committee, China Internet Network Information Center (2021). Research Report on Problematic Internet Use of Minors in China in 2020. http://china.cnr.cn/gdgg/20210720/t20210720_525539583.shtml.

[31] Cao H., Sun Y., Wan Y., Hao J., Tao F. (2011). Problematic Internet use in Chinese adolescents and its relation to psychosomatic symptoms and life satisfaction. BMC Public Health.

[32] Kożybska M., Szpak O., Kurpisz J., Lebiecka Z., Flaga-Gieruszyńska K., Samochowiec J., Karakiewicz B. (2019). Problematic Internet Use and health behaviors in adolescent residents of urban and rural areas in Poland–a cross-sectional study. Arch. Psychiatry Psychother..

[33] Jones C.N., You S., Furlong M.J. (2013). A preliminary examination of covitality as integrated well-being in college students. Soc. Indic. Res..

[34] Li Y., Zhao S., Li W., Liu H. (2021). Relationship Between Chinese Adolescents’ Sleep Status and Problem Behaviors: The Mediating Role of Mental Health. Front. Psychol..

[35] Wu Z.X. (2000). The Psychology and Prevention of Juvenile Delinquent Behavior.

[36] Chen X., Zhong H. (2012). Stress, negative emotions, and the deviant behaviors of Chinese migrant children. Issues Juv. Crimes Delinq..

[37] Sun R., Du P. (2020). Will parental participation affect the social behavior development of junior middle school students—The moderating effect of students’ gender and parents’ education level. Educ. Sci. Res..

[38] Fineberg N.A., Demetrovics Z., Stein D.J., Ioannidis K., Potenza M.N., Grünblatt E., Chamberlain S.R. (2018). Manifesto for a European research network into Problematic Usage of the Internet. Eur. Neuropsychopharmacol..

[39] Ha Y.M., Hwang W.J. (2014). Gender differences in internet addiction associated with psychological health indicators among adolescents using a national web-based survey. Int. J. Ment. Health Addict..

[40] Satici S.A., Uysal R. (2015). Well-being and problematic Facebook use. Comput. Hum. Behav..

[41] Fergus S., Zimmerman M.A. (2005). Adolescent resilience: A framework for. Annu. Rev. Public Health.

[42] Luthar S.S., Crossman E.J., Small P.J., Lamb M.E., Lerner R.M. (2015). Resilience and Adversity. Handbook of Child Psychology and Developmental Science: Socioemotional Processes.

[43] Dishion T.J., McMahon R.J. (1998). Parental monitoring and the prevention of child and adolescent problem behavior: A conceptual and empirical formulation. Clin. Child Fam. Psychol. Rev..

[44] Zhang Y., Chen Y., Zhang W. (2021). Community violence exposure and externalizing problem behavior among Chinese high school students: The moderating role of parental knowledge. Front. Psychol..

[45] Jiang Y., Yu C., Zhang W., Bao Z., Zhu J. (2016). Peer victimization and substance use in early adolescence: Influences of deviant peer affiliation and parental knowledge. J. Child Fam. Stud..

[46] McAdams T.A., Salekin R.T., Marti C.N., Lester W.S., Barker E.D. (2014). Co-occurrence of antisocial behavior and substance use: Testing for sex differences in the impact of older male friends, low parental knowledge and friends’ delinquency. J. Adolesc..

[47] Bronfenbrenner U. (1986). Ecology of the family as a context for human development: Research perspectives. Dev. Psychol..

[48] Chen Y., Zhang W., Zhu J., Yu C., Zhang Y., Lu Z. (2016). Peer victimization and problematic online game use among adolescents: A moderated mediation model. Psychol. Dev. Educ..

[49] Lahey B.B., Van Hulle C.A., D’Onofrio B.M., Rodgers J.L., Waldman I.D. (2008). Is parental knowledge of their adolescent offspring’s whereabouts and peer associations spuriously associated with offspring delinquency?. J. Abnorm. Child Psychol..

[50] Ang R.P., Chong W.H., Chye S., Huan V.S. (2012). Loneliness and generalized problematic Internet use: Parents’ perceived knowledge of adolescents’ online activities as a moderator. Comput. Hum. Behav..

[51] Zhang Y., Tan D.L., Lei T.T. (2020). Parental attachment and problematic smartphone use among Chinese young adults: A moderated mediation model of interpersonal adaptation and self-control. J. Adult Dev..

[52] Jantzer V., Haffner J., Parzer P., Resch F., Kaess M. (2015). Does parental monitoring moderate the relationship between bullying and adolescent nonsuicidal self-injury and suicidal behavior? A community-based self-report study of adolescents in Germany. BMC Public Health.

[53] Chang T.F., Qin D.B. (2018). Maternal monitoring knowledge change and adolescent externalizing behaviors in low-income African American and Latino families. Psychol. Rep..

[54] Stattin H., Kerr M. (2000). Parental monitoring: A reinterpretation. Child Dev..

[55] Kerr M., Stattin H., Trost K. (1999). To know you is to trust you: Parents’ trust is rooted in child disclosure of information. J. Adolesc..

[56] Crouter A.C., Bumpus M.F., Davis K.D., McHale S.M. (2005). How do parents learn about adolescents’ experiences? Implications for parental knowledge and adolescent risky behavior. Child Dev..

[57] Lin S., Yu C., Chen W., Tian Y., Zhang W. (2018). Peer victimization and aggressive behavior among Chinese adolescents: Delinquent peer affiliation as a mediator and parental knowledge as a moderator. Front. Psychol..

[58] Achenbach T.M. (1991). Manual for the Youth Self-Report and 1991 Profile.

[59] Achenbach T.M. (1991). Integrative Guide for the 1991 CBCL/4–18, YSR, and TRF Profiles.

[60] Feng L., Lan X. (2020). The moderating role of autonomy support profiles in the association between grit and externalizing problem behavior among family-bereaved adolescents. Front. Psychol..

[61] Xing X., Wang M., Zhang Q., He X., Zhang W. (2011). Gender differences in the reciprocal relationships between parental physical aggression and children’s externalizing problem behavior in China. J. Fam. Psychol..

[62] Li D., Zhang W., Li X., Zhen S., Wang Y. (2010). Stressful life events and problematic Internet use by adolescent females and males: A mediated moderation model. Comput. Hum. Behav..

[63] Young K.S. (1998). Internet addiction: The emergence of a new clinical disorder. Cyberpsychology Behav..

[64] Zhou Y., Li D., Li X., Wang Y., Zhao L. (2017). Big five personality and adolescent Internet addiction: The mediating role of coping style. Addict. Behav..

[65] McDowell I. (2006). Measuring Health: A Guide to Rating Scales and Questionnaires.

[66] Zhuo R., Yu Y., Shi X. (2022). Family Resilience and Adolescent Mental Health during COVID-19: A Moderated Mediation Model. Int. J. Environ. Res. Public Health.

[67] Sun P., Sun Y., Jiang H., Jia R., Li Z. (2019). Gratitude and problem behaviors in adolescents: The mediating roles of positive and negative coping styles. Front. Psychol..

[68] Wang Y., Fu C., Wang M. (2021). The additive and interactive effects of parental harsh discipline and boys’ gender-related traits on boys’ externalizing problem behaviors. Child. Youth Serv. Rev..

[69] Spada M.M. (2014). An overview of problematic Internet use. Addict. Behav..

[70] Tam P., Walter G. (2013). Problematic internet use in childhood and youth: Evolution of a 21st century affliction. Australas. Psychiatry.

[71] Donnerstein E., Strasburger V.C., Wilson B.J., Jordan A.B. (2009). The internet. Children, Adolescents, and the Media.

[72] Funk J.B., Strasburger V.C., Wilson B.J., Jordan A.B. (2009). Video games. Children, Adolescents, and the Media.

[73] Yu Q.J. (2020). Analysis of illegal and bad information on the Internet and adolescences’ deviant behavior. J. Guangxi Univ. Natl. Philos. Soc. Sci. Ed..

[74] Guan Y. (2013). Analysis of juvenile delinquency and its influencing factors—Based on the national survey of juvenile delinquents. Issues Juv. Crimes Delinq..

[75] Lan X., Wang W. (2020). To be Shy or avoidant? Exploring the longitudinal association between attachment and depressive symptoms among left-behind adolescents in rural China. Personal. Individ. Differ..

[76] Guo J., Zhu Y., Fang L., Zhang B., Liu D., Fu M., Wang X. (2020). The Relationship between Being Bullied and Addictive problematic Internet use among Chinese Rural Adolescents: The Mediating Effect of Adult Attachment. J. Interpers. Violence.

[77] Sung J., Lee J., Noh H.M., Park Y.S., Ahn E.J. (2013). Associations between the risk of internet addiction and problem behaviors among Korean adolescents. Korean J. Fam. Med..

[78] Laukkanen E., Shemeikka S., Notkola I.L., Koivumaa-Honkanen H., Nissinen A. (2002). Externalizing and internalizing problems at school as signs of health-damaging behaviour and incipient marginalization. Health Promot. Int..

[79] Beranuy M., Carbonell X., Griffiths M.D. (2013). A qualitative analysis of online gaming addicts in treatment. Int. J. Ment. Health Addict..

[80] Arora B. (2016). Exploring and analyzing Internet crimes and their behaviours. Perspect. Sci..

[81] Kor A., Zilcha-Mano S., Fogel Y.A., Mikulincer M., Reid R.C., Potenza M.N. (2014). Psychometric development of the problematic pornography use scale. Addict. Behav..

[82] Lazuras L., Barkoukis V., Tsorbatzoudis H. (2017). Face-to-face bullying and cyberbullying in adolescents: Trans-contextual effects and role overlap. Technol. Soc..

[83] O’Gara J.L., Zhang A., Padilla Y., Liu C., Wang K. (2019). Father-youth closeness and adolescent self-rated health: The mediating role of mental health. Child. Youth Serv. Rev..

[84] Tian Y., Yu C., Lin S., Lu J., Liu Y., Zhang W. (2019). Sensation seeking, deviant peer affiliation, and internet gaming addiction among Chinese adolescents: The moderating effect of parental knowledge. Front. Psychol..

